# Effects of micronized progesterone added to non-oral estradiol on lipids and cardiovascular risk factors in early postmenopause: a clinical trial

**DOI:** 10.1186/1476-511X-11-133

**Published:** 2012-10-09

**Authors:** Gislaine Casanova, Poli Mara Spritzer

**Affiliations:** 1 Gynecological Endocrinology Unit, Division of Endocrinology, Hospital de Clínicas de Porto Alegre (HCPA), Rua Ramiro Barcelos, 2350, 90035-003, Porto Alegre, Brazil; 2 National Institute of Hormones and Women’s Health, CNPq, Rua Ramiro Barcelos, 2350, 90035-003, Porto Alegre, Brazil; 3 Laboratory of Molecular Endocrinology, Department of Physiology, Universidade Federal do Rio Grande do Sul (UFRGS), Rua Ramiro Barcelos, 2350, 90035-003, Porto Alegre, Brazil

**Keywords:** Lipid profile, Early postmenopause, Micronized progesterone, Non-oral estrogen, Hormone therapy

## Abstract

**Background:**

Much attention has been drawn to the deleterious effects of adding progestins to estrogen as hormone therapy (HT) in postmenopausal women. Some widely prescribed progestins have been shown to partially oppose the beneficial effects of estrogens on surrogate markers of cardiovascular disease (CVD) risk. Progestins with higher androgenic activity may interfere with lipid profile and glucose tolerance, and could affect mechanisms of estrogen-induced C-reactive protein (CRP) stimulation. Recent data have shown that norpregnane derivatives, but not micronized progesterone, increase the risk of venous thromboembolism among transdermal estrogens users. The aim of the present study was to assess the effects of combining micronized progesterone with non-oral estrogen therapy on lipid profile and cardiovascular risk factors in a sample of early postmenopausal women.

**Methods:**

Clinical trial including 40 women receiving intranasal 17β estradiol 3 mg/day for two months and 46 women receiving percutaneous 17β estradiol gel 1.5 mg/day for three months (E2). Both groups received an additional 200 mg/day of micronized progesterone by vaginal route 14 days/month (E2+P). Outcome measures included body weight, waist circumference, body mass index (BMI), lipid profile and ultra-sensitive C-reactive protein (usCRP) at baseline and during the E2 or E2+P portions of treatment.

**Results:**

Mean age was 51±3 years. Mean time since menopause was 22.2±10 months. Most participants were overweight; HT did not change BMI. E2 and E2+P did not affect waist circumference and weight. Menopausal symptoms improved after HT. The effects of intranasal and percutaneous estradiol were similar, regardless of the addition of progesterone. Similarly, for the overall group of 86 women, micronized progesterone did not alter the response to E2. Blood pressure, glucose, insulin, HDL-c, triglycerides, and usCRP remained constant with or without micronized progesterone. Total cholesterol decreased after E2, and progesterone maintained this reduction. LDL-c levels were similar at baseline and with E2, and lower during E2+P in relation to baseline.

**Conclusions:**

Cyclic, short term exposure to vaginal micronized progesterone did not alter the metabolic and cardiovascular effects of non-oral E2 in early, apparently healthy, postmenopausal women.

**Trial registration:**

ClinicalTrials.gov NCT01432028

## Background

Much attention has been drawn to the deleterious effects of adding progestins to estrogen as hormone therapy (HT) in postmenopausal women [1]. Recent prospective randomized studies have raised great concern regarding this combination, which has been linked to a negative impact on the cardiovascular and venous systems and on cognition [2], as well as to the development of breast cancer [3] in women in the menopause transition and postmenopause.

Progestogens encompass both progesterone, the physiological molecule synthesized and secreted by the ovary, and synthetic compounds named progestins [4]. All progestins share a progestogenic effect that causes the endometrium to enter the secretory phase and determines a decrease in endometrial disease [5]. However, other biological effects of progestins vary widely, since each progestin or progestin metabolite binds to specific steroid receptors. Some of the most widely prescribed progestins have been shown to partially oppose the beneficial effects of estrogens on surrogate markers of cardiovascular disease (CVD) risk [4]. Progestins with higher androgenic activity may interfere with lipid profile and glucose tolerance [6], and could affect mechanisms of estrogen-induced C-reactive protein (CRP) stimulation [7].

Even though the route of estrogen administration is known to be an important determinant of cardiovascular risk in postmenopausal women using HT [8], recent data have shown that norpregnane derivatives, but not micronized progesterone, increase the risk of venous thromboembolism among transdermal estrogens users [9]. A few studies indicate that micronized progesterone may have a better risk profile with respect to variables related to cardiovascular risk [10,11]. Therefore, not only the route of estrogen administration, but also the type of progestin may be important in determining the overall benefit-risk ratio for HT.

The aim of the present study was to assess the effects of combining natural micronized progesterone with non-oral estrogen therapy on variables related to lipid and hormonal profile and on ultra-sensitive C-reactive protein (usCRP) in a sample of early postmenopausal women.

## Results

The mean age of participants was 51 ± 3 years, and 96% were Caucasian (the remaining 4% were of mixed African and European ancestry). Mean age at menopause was 49.4 ± 3 years, and mean time since menopause was 22.2 ± 10 months. Thirteen (15%) patients were smokers.

The effects of intranasal and percutaneous gel were similar during E2 and E2+P (Table 1). Table 2 presents body mass index (BMI), weight, waist circumference, blood pressure and Kupperman score for menopausal symptoms before and during E2 and E2+P in the overall group of 86 participants. Most participants were overweight. BMI did not change with HT. Similarly, waist circumference, weight and systolic and diastolic blood pressure remained unchanged during HT with E2 alone or E2+P. At baseline, all patients presented menopausal symptoms that improved significantly with treatment, as shown by the Kupperman score (Table 2).

**Table 1:** Anthropometric and clinical variables according to type of non-oral estradiol (intranasal or percutaneous gel)

	**Baseline**		**E2**		**E2+P**		***P***
	**Intranasal**	**Percutaneous**	**Intranasal**	**Percutaneous**	**Intranasal**	**Percutaneous**	
	**n = 40**	**n = 46**	**n = 40**	**n = 46**	**n = 40**	**n = 46**	
BMI	26 ± 3	26 ± 3	26 ± 3	26 ± 3	26 ± 3	26 ± 3	0.6
Weight (kg)	66 ± 7	64 ± 9	66 ± 7	64 ± 9	66 ± 7	64 ± 10	0.6
WC (cm)	84 ± 6	84 ± 8	84 ± 6	84 ± 8	83.5 ± 5	83.6 ± 9	0.6
SBP (mmHg)	118 ± 15	119 ± 12	116 ± 15	114 ± 15	118 ± 14	116 ± 14	0.4
DBP (mmHg)	75 ± 7	77 ± 7	75 ± 9	74 ± 9	76 ± 10	74 ± 10	0.2
Total-c (mg/dL)	222 ± 31^a†^	211 ± 27^a†^	212 ± 31^b†^	201 ± 27^b†^	205 ± 31^b†^	200 ± 32^b†^	0.5
HDL-c (mg/dL)	63 ± 12	63 ± 18	62 ± 14	60 ± 15	63 ± 14	60 ± 16	0.2
LDL-c (mg/dL)	134 ± 28^a†^	125 ± 29^a†^	128 ± 32^a,b†^	118 ± 26^a,b†^	121 ± 26^b†^	117 ± 29^b†^	0.5
Triglycerides (mg/dL)	122 ± 49	117 ± 56	117 ± 48	114 ± 54	108 ± 42	115 ± 62	0.3
Fast glucose (mg/dL)	91 ± 11	92 ± 9	91 ± 8	91 ± 8	93 ± 11	91 ± 10	0.2
2h glucose (mg/dL)	106 ± 29	102 ± 23	113 ± 38	103 ± 35	110 ± 39	101 ± 31	0.2
Insulin (μU/mL)	7 (4–10)	6 (3–9)	6 (2–8)	7 (3–9)	6 (4–9)	7 (3–9)	0.2
Estradiol (pg/mL)	14 (8–17)	11 (5–20)	40 (9–121)	65 (20–119)	47 ( 13–68)	49 (10–96)	0.1
hsCRP (mg/L)	2.1 (0.4-3.9)	1.1 (0.3-2.3)	1.6 (0.5-3)	1.4 (0.2-3)	1.5 (0.5-2)	1 (0.2-2)	0.6

Values expressed as median and interquartile range or mean ± SD. Two-way analysis of variance with repeated measures (non parametric variables were log-converted for statistical analysis and reconverted for presentation in table format). P = difference between intranasal and percutaneous treatment.

† = P < 0.01 for difference between baseline, E2 and E2+P. Different superscript letters indicate statistical difference with Bonferroni multiple-comparison correction test (α < 5%).

Baseline: before hormone therapy; E2: estradiol only; E2+P estradiol + micronized progesterone.

Intranasal: 3 mg/day 17β estradiol by intranasal route (n = 40) for two months. Percutaneous: 1.5 mg/day 17β estradiol gel by percutaneous route (n = 53) for three months.

*BMI* body mass index, *DBP* diastolic blood pressure, *HDL-c* high-density lipoprotein cholesterol, *hsCRP* high-sensitivity C-reactive protein test, *LDL-c* low-density lipoprotein cholesterol, *SBP* systolic blood pressure, *Total-c* total lipoprotein cholesterol, *WC* waist circumference, 2h glucose: glucose levels 2 hours after 75g oral glucose load.

**Table 2:** Clinical and anthropometric variables and menopausal symptoms (n = 86)

	**Baseline**	**E2**	**E2+P**	***P***
BMI	26.3 ± 3	26.29 ± 3	26.23 ± 3.1	0.8
Weight (kg)	65.5 ± 8.3	65.58 ± 8.3	65.4 ± 8.6	0.7
Waist circumference (cm)	84.3 ± 7.5	84.2 ± 7.6	83.5 ± 7.4	0.06
Systolic blood pressure	118.6 ± 13.5	115 ± 14.9	116.7 ± 14.3	0.1
Diastolic blood pressure	76.2 ± 7.3	74.7 ± 9.5	74.9 ± 9.7	0.4
Kupperman score	26 (17–30)^a^	6 (0–12.5)^b^	4 (0–8)^b^	< 0.01
E2 (pg/mL)	13 (5–19)^a^	54 (13–122)^b^	48 (13–80)^b^	< 0.01

Values expressed as median and interquartile range or mean ± SD. P = two-way analysis of variance with repeated measures (non parametric variables were log-converted for statistical analysis and reconverted for presentation in table format).

Different superscript letters indicate statistical difference with Bonferroni multiple-comparison correction test (α < 5%).

Baseline: before hormone therapy; E2: estradiol only; E2+P estradiol + micronized progesterone.

*BMI* body mass index.

Table 3 shows metabolic variables and usCRP at baseline and during treatment for the overall group. Glucose, insulin, high-density lipoprotein cholesterol (HDL-c), triglycerides, and the high-sensitivity C-reactive protein test (hsCRP) remained constant after non-oral therapy with or without micronized progesterone. Total cholesterol decreased after E2-only treatment, and the addition of progesterone maintained this reduction. Low-density lipoprotein cholesterol (LDL-c) levels were similar at baseline and with E2 only, and were lower during E2+P treatment in relation to baseline.

**Table 3:** Anthropometric and metabolic variables and markers of endothelial function (n = 86)

	**Baseline**	**E2**	**E2+P**	***P***
Total-c (mg/dL)	216 ± 31^a^	207 ± 30^b^	203 ± 32^b^	< 0.01
HDL-c (mg/dL)	63 ± 16	61 ± 15	61 ± 15	0.06
LDL-c (mg/dL)	129 ± 29^a^	123 ± 29^a,b^	119 ± 28^b^	< 0.01
Triglycerides (mg/dL)	120 ± 53	115 ± 51	111 ± 53	0.2
Fast glucose (mg/dL)	91 ± 11	91 ± 8	92 ± 11	0.2
2h glucose (mg/dL)	103 ± 27	108 ± 36	105 ± 36	0.2
Insulin (μU/mL)	6.9 (3.9-9.7)	6.2 (2.8 -8.8)	6.9 (3.8 -9.1)	0.2
hsCRP (mg/L)	1.51 (0.38-2.9)	1.64 (0.4-3)	1.19 (0.3-2.1)	0.1

Values expressed as median and interquartile range or mean ± SD. P = two-way analysis of variance with repeated measures (non parametric variables were log-converted for statistical analysis and reconverted for presentation in table format).

Different superscript letters indicate statistical difference with Bonferroni multiple-comparison correction test (α < 5%).

Baseline: before hormone therapy; E2: estradiol only; E2+P estradiol + micronized progesterone.

*HDL-c* high-density lipoprotein cholesterol, *hsCRP* high-sensitivity C-reactive protein test, *LDL-c* low-density lipoprotein cholesterol, *Total-c* total lipoprotein cholesterol; 2h glucose: glucose levels 2 hours after 75g oral glucose load.

## Discussion

In the present study, cyclic exposure to vaginally administered micronized progesterone over the short term failed to affect lipid profile in early and apparently healthy postmenopausal women. Several studies have evaluated the relationship between estrogen dose and/or route of administration and cardiovascular benefit-risk ratio of HT in postmenopausal women. More recently, observational studies began to draw attention to the impact of using specific types of progestin in combination with estrogen. However, there is a paucity of data derived from clinical trials to assess the effect of different progestogens on variables related to cardiovascular risk. Therefore, this work provides an important contribution toward clarifying the impact of combined micronized progesterone plus non-oral estrogen therapy.

Menopause is a risk factor for CVD because of the ensuing endogenous estrogen deficiency, which has a detrimental effect on cardiovascular function and metabolism. Even though there are biologically plausible mechanisms of cardiovascular protection against harm produced by estrogen therapy, recent clinical trials suggest that estrogen may be associated with cardiovascular risk rather than benefit in the postmenopause [12]. However, reanalysis of these studies has indicated a possible protective window in which recent postmenopausal women in their sixth decade may benefit from HT [1,13]. In addition, it has been speculated that the cardioprotective benefits of HT may be more evident in the early postmenopausal period [14], although this is a controversial issue [15]. In the present study, the use of a sample of apparently healthy and relatively young (mean age of 51.3 ± 3 years) women who were postmenopausal for less than three years (22.2 ± 10 months) enabled us to more accurately demonstrate the neutral or beneficial effects of HT with both non-oral E2 alone or in combination with micronized progesterone- combined period.

Blood pressure levels remained unchanged after HT in the present sample. Previous studies have shown that non-oral estrogen therapy associated with micronized progesterone had no deleterious effects on blood pressure in normotensive and controlled hypertensive postmenopausal women [10,16]. Estrogen increases the release of nitric oxide causing relaxation of smooth muscle cells and vasodilatation.

Progestins modulate the effects of estrogen on hepatic endocrine function through intrinsic androgenic properties. When co-administered with estrogen, progestogen may also have significant effects on body composition and metabolism because of its androgenic properties. The effects of HT on weight and body composition remain controversial [17]. In our study, non-oral E2 therapy with or without micronized progesterone did not modify waist circumference, BMI or body weight. Additionally, the treatment did not interfere with glucose or insulin levels, and reduced total cholesterol and LDL-c, supporting the notion that micronized progesterone has a neutral effect on intermediate surrogate variables of cardiovascular risk [18]. Dansuk et al. [19] evaluated the effects of five combinations of HT in postmenopausal women, including E2 alone and E2 associated with medroxyprogesterone (E2+MPA), noretisterone (E2+NETA), dydrogesterone (E2+DG) and micronized progesterone (E2+P). E2+NETA and E2+DG were found to improve insulin sensitivity after 3 months of treatment, whereas E2+P or E2 alone did not show such any effect in postmenopausal women.

It is well established that oral E2 therapy in conventional doses induces an increase in hsCRP, while transdermal E2 has either no effect on or even reduces hsCRP levels in postmenopausal women [20]. Studies examining the effect of progestins on markers of inflammation have produced varying results [18]. In the present study there was no worsening of hsCRP during HT with or without progesterone.

A limitation of this study is the short duration of treatment (6 months or less), since in clinical practice patients are usually treated for one year or more. Nevertheless, previous studies have reported significant changes in lipids and markers of endothelial function [21] after 4 to 12 weeks of HT. In addition, evidence suggests that there is a “critical period” in the first months of HT, related to greater inflammatory activation and higher thromboembolic risk events. Further studies of longer duration will be helpful to confirm our findings.

## Conclusions

Data from the present study suggest that the addition of micronized progesterone to non-oral E2 did not induce harmful effects on variables related to cardiovascular risk in a population of healthy, early postmenopausal women. Micronized progesterone did not interfere with the effects of non-oral E2, and did not abrogate the relief of symptoms. Finally, combined micronized progesterone and non-oral E2 treatment had neutral impact on blood pressure, body composition, lipid profile and markers of endothelial function.

## Methods

### Study protocol

This study is nested within a crossover randomized trial assessing the comparative effects of low-dose oral HT and non-oral HT on cardiovascular risk factors and markers of endothelial function in early postmenopausal women. Preliminary results of this trial (which focused on the comparison between non-oral estradiol-micronized progesterone or low-dose oral estradiol-drospirenone therapy on metabolic variables and markers of endothelial function in early postmenopause), including the first 40 women enrolled, have been published [22].

In the present analysis, 42 women received 3 mg/day 17β estradiol by intranasal route (Aerodiol®, Servier, RJ, Brazil) for two months, and 53 received 1.5 mg/day 17β estradiol gel by percutaneous route (Oestrogel®, Farmoquímica, SP, Brazil) for three months. Additionally, both groups received 200 mg/day micronized progesterone (Utrogestan®, Farmoquímica, SP, Brazil) together with estrogen treatment by vaginal route 14 days/month during the studied cycles (during 2 or three months, respectively). The following were compared: effects of type of non-oral estradiol (intranasal or percutaneous gel) before, during the estradiol only portion of the study (E2) and during the estradiol plus progesterone portion (E2+P). The effects of E2 vs. E2+P for the overall group, regardless of route (intranasal or percutaneous), were also analyzed.

The 95 women enrolled for this trial fulfilled the following inclusion criteria: 1) last menstrual period between 6 months and 3 years before the beginning of the study plus follicle-stimulating hormone (FSH) levels higher than 35 IU/L; 2) age between 42 and 58 years; 3) no use of any medication known to interfere with hormonal, glucose or lipoprotein levels in the past 3 months; 4) no use of steroidal or non-steroidal anti-inflammatory drugs in the last 15 days. Patients presenting diabetes, previous hysterectomy, endometrial thickness higher than 0.5 cm, history of cancer, thromboembolism or established CVD were excluded.

**Figure 1:**
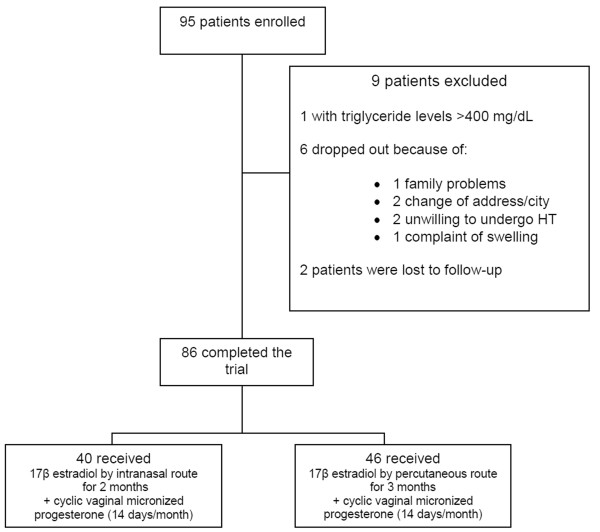
**Representation of the design.** Flowchart showing the total number of patients enrolled, number of dropouts, and group description.

Nine patients dropped out in the first two months of follow-up (Figure 1). Therefore, 86 patients completed the study. Clinical evaluation was performed before the treatment was begun and monthly during the trial. Anthropometric measurements included body weight, height, waist circumference (measured at the midpoint between the lower rib margin and the iliac crest), hip circumference (measured at the level of the greater trochanter), waist-to-hip ratio (WHR), and BMI, current measured weight in kg divided by height in m^2^). The Kupperman score was assessed before and during treatment. Blood pressure was measured twice, at a 1-min interval in seated patients, using a digital sphygmomanometer (Omron HEM 742, Rio de Janeiro, Brazil) with appropriate cuff for the arm diameter.

The study protocol was approved by the Ethics Committee at the Hospital de Clínicas de Porto Alegre, and written informed consent was obtained from every subject (IRB 0000921/05-053). The study was registered at clinicaltrials.gov (NCT01432028).

### Laboratory assessment

Blood samples were collected before treatment, during the estradiol-only portion (E2) (days 12 to 14 of the second treatment month), and at the end of treatment (days 24 to 28 of the last month of estradiol plus micronized progesterone administration) (E2+P). All samples were obtained between 08:00 and 10:00 a.m. After a 12-hour overnight fast, blood samples were drawn from an antecubital vein for determination of FSH, estradiol (E2), lipid profile (total cholesterol, HDL-c and triglycerides) plasma glucose (oral glucose tolerance test), and insulin. Blood samples were also drawn for us-CRP.

Total cholesterol, HDL-c, triglycerides, and glucose were determined by colorimetric-enzymatic methods using the Bayer 1650 Advia System (Mannheim, Germany). LDL-c was estimated indirectly using the formula LDL = total cholesterol - HDL-c - triglycerides / 5.

Serum FSH was measured by electrochemiluminescence immunoassay (ECLIA), with intra and interassay coefficients of variation (CV) of 1.8% and 3.3% for FSH. The sensitivity of the assay was 0.05 IU/L for FSH. Estradiol was measured by ECLIA (Roche Diagnostics, Mannheim, Germany), with an assay sensitivity of 5.0 pg/mL and intra and interassay CV of 5.7 and 6.4%. Serum insulin levels were measured using ECLIA (Roche Diagnostics, Mannheim, Germany), with sensitivity of 0.200 μIU/mL and intra and interassay CV of 2.0 and 4.3%, respectively. Ultra-sensitive CRP was assayed using stored specimens, with a validated high-sensitivity nephelometric method (Dade Behring Marburg, Marburg, Germany). Sensitivity was 0.17 mg/L and intra and interassay CV were 4.4 and 5.7%, respectively. For data analysis, individual results below the limit of sensitivity were considered as equal to 0.17 mg/L.

### Statistical analysis

Results are expressed as means ± standard deviation (SD) or median and interquartile range. Log10 transformation was used to normalize the distribution of non-Gaussian variables. Two-way analysis of variance (ANOVA) with repeated measures was carried out for comparing basal conditions, E2 and E2+P. Bonferroni adjustment was used for multiple comparisons. Friedman test was used for the analysis of Kupperman score, followed by the sign test. All analyses were performed using the Statistical Package for the Social Sciences (SPSS, Chicago, IL, USA) and Stata (StataCorp LP, Texas, USA). Data were considered to be significant at P < 0.05.

## Abbreviations

ANOVA: Analysis of variance; BMI: Body mass index; CRP: C-reactive protein; CV: Coefficients of variation; CVD: Cardiovascular risk; DBP: Diastolic blood pressure; DG: Dydrogesterone; E2: Estradiol; ECLIA: Electrochemiluminescence immunoassay; FSH: Follicle-stimulating hormone; HDL-c: High-density lipoprotein cholesterol; hsCRP: High-sensitivity C-reactive protein test; HT: Hormone therapy; LDL-c: Low-density lipoprotein cholesterol; MPA: Medroxyprogesterone; NETA: Noretisterone; P: Progesterone; SBP: Systolic blood pressure; SD: Standard deviation; SPSS: Statistical Package for the Social Sciences; usCRP: Ultra-sensitive C-reactive protein; WC: Waist circumference; WHR: Waist-to-hip ratio.

## Competing interests

The authors declare that they have no competing interests.

## Authors' contributions

GC and PMS contributed to acquisition of data, analysis and interpretation of data and manuscript review. PMS conceived and designed the study. Both authors contributed to the analysis and interpretation of data, drafting manuscript and final review, and both approved the final version of the manuscript.
